# Room-temperature subnanosecond waveguide lasers in Nd:YVO_4_ Q-switched by phase-change VO_2_: A comparison with 2D materials

**DOI:** 10.1038/srep46162

**Published:** 2017-04-06

**Authors:** Weijie Nie, Rang Li, Chen Cheng, Yanxue Chen, Qingming Lu, Carolina Romero, Javier R. Vázquez de Aldana, Xiaotao Hao, Feng Chen

**Affiliations:** 1 School of Physics, State Key Laboratory of Crystal Materials, and Key Laboratory of Particle Physics and Particle Irradiation (Ministry of Education), Shandong University, 250100 Jinan, Shandong, China; 2 School of Chemistry and Chemical Engineering, Shandong University, Jinan 250100, China; 3 Laser Microprocessing Group, Universidad de Salamanca, 37008 Salamanca, Spain

## Abstract

We report on room-temperature subnanosecond waveguide laser operation at 1064 nm in a Nd:YVO_4_ crystal waveguide through Q-switching of phase-change nanomaterial vanadium dioxide (VO_2_). The unique feature of VO_2_ nanomaterial from the insulating to metallic phases offers low-saturation-intensity nonlinear absorptions of light for subnanosecond pulse generation. The low-loss waveguide is fabricated by using the femtosecond laser writing with depressed cladding geometry. Under optical pump at 808 nm, efficient pulsed laser has been achieved in the Nd:YVO_4_ waveguide, reaching minimum pulse duration of 690 ps and maximum output average power of 66.7 mW. To compare the Q-switched laser performances by VO_2_ saturable absorber with those based on two-dimensional materials, the 1064-nm laser pulses have been realized in the same waveguide platform with either graphene or transition metal dichalcogenide (in this work, WS_2_) coated mirror. The results on 2D material Q-switched waveguide lasers have shown that the shortest pulses are with 22-ns duration, whilst the maximum output average powers reach ~161.9 mW. This work shows the obvious difference on the lasing properties based on phase-change material and 2D materials, and suggests potential applications of VO_2_ as low-cost saturable absorber for subnanosecond laser generation.

Waveguide lasers are miniature light sources that could be applied in integrated photonic systems to achieve various applications^1^,^2^,^3^,^4^,^5^,^6^. By using suitable techniques, low-loss waveguides can be produced in laser crystals with diverse geometries, offering cost-effective platforms for solid-state lasing generation in compact volumes. Benefited from the high intracavity optical intensities, the waveguide lasing could be generated at much low thresholds with comparable efficiencies to bulk and fiber laser systems^7^,^8^,^9^. Recently, it has been proved that with specially designed photonic structures, waveguide lasing can be generated for tailored beam evolution with modulated modal profiles, e.g., beam splitting or ring-shaped transformation^10^,^11^. These features enable waveguide lasers intriguing applications as miniature light sources in the integrated photonic circuits.

The first significant step to implement waveguide laser systems is to fabricate low-loss waveguide in laser gain media. A number of techniques have been developed diverse active waveguide structures in crystalline materials^4^,^12^,^13^,^14^. As a powerful 3D micro-nano fabrication technique, femtosecond laser writing/inscription has been widely applied to produce various photonic devices or components in optical materials^15^,^16^,^17^,^18^,^19^. Femtosecond laser written dielectric waveguide laser systems have been realized in a number of active glasses, ceramics and crystals, offering broadband laser operations from visible to mid-infrared (MIR) wavelength regimes^20^,^21^,^22^,^23^. Additionally, the advantage of laser inscription technology for direct-write 3D waveguide fabrication enables designed laser beam tailoring at direct optical pump, which shows superior features on the laser shaping to non-direct-write waveguide laser systems^10^,^11^,^22^. Lasing in waveguides, similar to the bulk systems, can be realized in continuous wave (CW) as well as pulsed operation regimes. The performance of crystalline waveguide lasers depends on the substrate materials and the waveguiding geometries. For example, the CW waveguide laser produced by femtosecond laser writing has reached a maximum output power as high as ~5 W (in a Yb:YAG Type II double-line waveguide platform) at 1030 nm, whilst only vertical polarized modes (i.e., TM modes) are guided^23^. However, in Type II double-line vanadate waveguides, e.g., Nd:YVO_4_ and Nd:GdVO_4_, the waveguide lasing operates at both TE and TM polarizations^24^,^25^,^26^.

Recently, waveguide lasers operating at pulsed regimes have attracted much more attentions due to the feasibility of on-chip nonlinear optical applications. The passive Q-switching or mode-locking are intriguing for implementation of pulsed waveguiding laser systems. By using suitable saturable absorbers (SAs), including Cr ion doped crystals, semiconductor saturable absorber mirror (SESAM), single-wall carbon nanotubes (SWCNT), 2D materials (e.g., graphene, black phosphorus, and transition metal dichalcogenides (TMDC)), laser pulses in laser-written crystalline waveguide systems have been realized in near infrared wavelength bands^27^,^28^,^29^,^30^,^31^,^32^,^33^,^34^,^35^,^36^,^37^,^38^. As compared to mode-locked lasers, the Q-switched systems possess advantages of higher output powers but the limitation is the relatively long pulse duration. Most results on waveguide lasing by passive Q-switching through the 2D materials and other SAs are in the timescale of nanosecond to microsecond regimes^39^,^40^,^41^. This may be partly due to the low-intracavity power of the waveguide laser, which is below the saturation intensity of most SAs. More recently, the nanomaterial of VO_2_ has shown its unique nonlinear optical properties during the phase-change process^42^,^43^. Owing to ultrafast phase transition to manipulate the transmittance of the light and large difference of optical nonlinearities between the two states of phase-transition materials (VO_2_), the VO_2_ therefore shows SA-like properties that could be utilized for Q-switching. The origin of special light absorption is due to the structural changes of VO_2_ from monoclinic (insulating) to tetragonal (metallic) phase^44^. Tan *et al*. reported on tunable waveguide lasers on carbon ion implanted Nd:YAG waveguides with dynamic thermal controlling of the VO_2_ mirror^45^. This mechanism is disparate with the SA of 2D materials based on photon-induced electronic excitation according to their band structures^46^. From technical point of view, a direct comparison of Q-switched waveguide lasers through SA of VO_2_ and the 2D materials is crucial for exploration on different effects of those nanomaterials on pulsed laser generation in compact, cost-effective photonic platforms. In this work, we apply a laser-written Nd:YVO_4_ waveguide as the laser cavity, and implement Q-switching by VO_2_, graphene, and WS_2_ SAs, respectively. A symmetric comparison on the pulsed laser performances on the three SA-based waveguide systems has been performed at same pump and operation conditions.

## Results

### Waveguide characteristics

**Figure 1:**
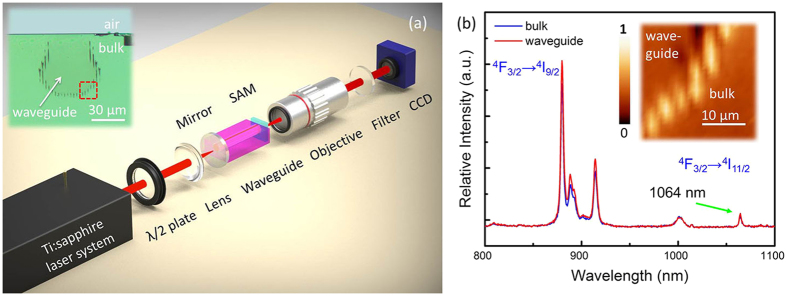
The fabrication and fluorescence characteristic of superficial cladding waveguides. (**a**) Schematic of experimental arrangement for the passively Q-switched laser operation in Nd:YVO_4_ superficial cladding waveguide. The insert is the microscope image of the laser-written waveguide. (**b**) Room-temperature fluorescence emission spectra (μPL) related to the ^4^F_3/2_ → ^4^I_9/2_ and ^4^F_3/2_ → ^4^I_11/2_ transitions of Nd^3+^ ions obtained from the laser inscribed waveguide volume (red solid line) and bulk region (blue solid line) of Nd:YVO_4_ crystal. The insert in (**b**) shows the 2D spatial distribution of the emitted intensity of Nd^3+^ emission line obtained from the end-face of waveguide, which corresponds to the region within the red dashed square in the microscope image (i.e., the insert of **a**).

Figure 1(a) exhibits the process of room-temperature Q-switched waveguide laser generation, and the optical microscope cross-sectional image of waveguide is shown in the inset. The structure consists of an unmodified core surrounded by major arc geometry of parallel low-index tracks and the interface of sample and air, forming a superficial cladding shape. Following the desired geometry with a lateral separation of 3 μm between each two adjacent damage tracks, the superficial cladding waveguide with the diameter of 50 μm is achieved. Compared with the applied buried configurations, this surface cladding waveguide may be more suitable for Q-switched pulse laser system since low intracavity losses can be easily introduced based on the evanescent-field interaction of surface coated SAs. In addition, it retains some advantages of cladding waveguide which can support waveguide lasers at both TM and TE polarizations and be used to construct fiber-waveguide-fiber integrated photonic chips with high coupling efficiency. Figure 1(b) demonstrates the comparison of the μ-PL emission spectra of Nd^3+^ ions related to the ^4^F_3/2_ → ^4^I_9/2_ and ^4^F_3/2_ → ^4^I_11/2_ emission lines from the bulk and the waveguide core. As it can be seen, both of them are virtually similar, manifesting that the fluorescence properties of Nd^3+^ ions at the waveguide volume have been well-preserved after laser writing procedure, exhibiting the potential applications as the gain medium of integrated laser sources. In order to obtain detailed information in the spatial extension of the variation observed in the Nd^3+^ luminescence, the spatial distribution map of the emission intensity of the ^4^F_3/2_ → ^4^I_9/2_ transition is measured as displayed in the insert of Fig. 1(b). The scanning area covering the modified and unmodified Nd:YVO_4_ volume corresponds to the region within the red dashed square in the microscope image (i.e., the insert of Fig. 1(a)). We can clearly see that local modifications have been induced by the femtosecond laser pulses, revealing the damage creation of the microstructural lattices. This results in the reduction of the refractive index in the laser induced tracks to form a low-index cladding which confines light inside the waveguide structure. It should be mentioned that, the 2D map also denotes a good preservation of fluorescence efficiency at the waveguide volume.

**Figure 2:**
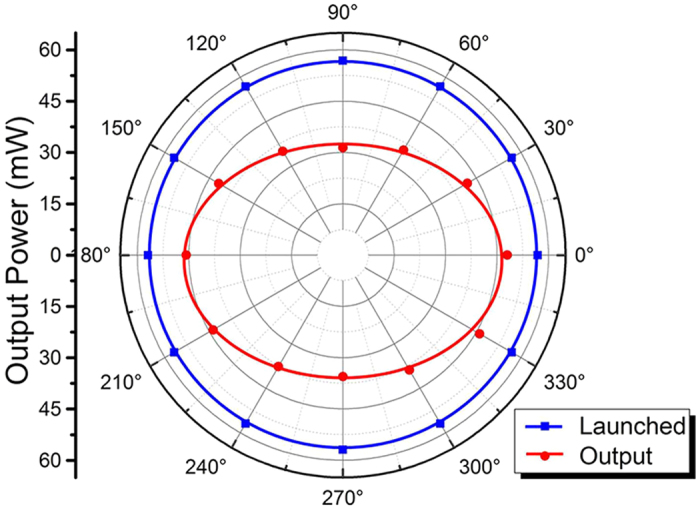
Polarization properties of superficial waveguide. Measured Transmitted power (red line) of superficial cladding waveguide versus all-angle light transmission with the same launched pump power (blue line) at 1064 nm.

To obtain the polarization effect on guided light transmission, the all-angle light guidance along the transverse plane is measured and displayed in the Fig. 2 at the wavelength of 1064 nm. With the same launched power at 56.88 mW, the output power at transverse electric (TE) polarization (i.e., 0°) is slightly larger than that at transverse magnetic (TM) polarization (i.e., 90°), which is varied smoothly with the change of launched beam polarization. The result may be owing to the synergy effect of the noncentrosymmetrical waveguide structure and the crystal anisotropy. Nevertheless, the guiding characteristic of the surface cladding waveguide still shows relative low sensitivity to polarization of the light. This feature is better than the reported simple dual-line waveguides fabricated in other crystals (e.g., Nd:YAG and Nd:GGG) only supporting the TM-guided modes^47^. In addition, the propagation losses, as one of the most crucial factors for waveguide quality, are measured and calculated to be ~0.25 dB/cm and ~0.82 dB/cm along TE and TM polarization, respectively. Eventually, the waveguide has been ascertained to be well-preserved fluorescence characteristic and low-loss guiding properties, which offers the possibility of the implementation of the pulsed waveguide lasers.

### Q-switched waveguide lasers

Based on the low-loss Nd:YVO_4_ waveguide, the passively Q-switched waveguide laser has been realized with VO_2_ SAM, which is the first time to achieve the subnanosecond pulse duration in a simple waveguide platform at room temperature (i.e., without assistance of any temperature control system). The short pulse duration enables to concentrate the laser energy on an extremely short time, resulting in the high peak power generation. In practice, it is on demand for many applications such as super-resolution microscopy bio-imaging and advanced materials processing^48^,^49^. It should be pointed out that the maximum pump power in our work is below the threshold of VO_2_ phase transition at the room temperature. In other words, the VO_2_ keeps the low-temperature insulating phase with a monoclinic crystal system. In comparison to the laser performances with the 2D materials based systems, the 1064-nm laser pulses are implemented with multilayer graphene and WS_2_ film coated SAMs using the same waveguide platform, too.

**Figure 3:**
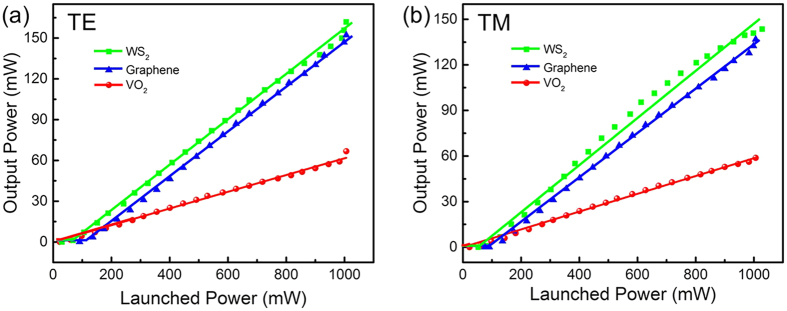
The average output power of pulsed laser with different SAs. The average output power at 1064 nm as a function of launched pump power in pulsed regime with SAMs of VO_2_ (red line), graphene (blue line) and WS_2_ (green line) along (**a**) TE and (**b**) TM polarization, respectively.

Figures 3(a) and (b) show the average output powers at 1064 nm as a function of launched pump power in pulsed regime with three different SAMs along TE and TM polarization, respectively. The fitted straight lines with the color of red, blue and green denote the pulse laser performance with VO_2_, graphene, and WS_2_ SAMs, separately. For TE polarization (Fig. 3(a)), the pulsed waveguide laser oscillation begins with VO_2_, graphene, and WS_2_ SAMs when the launched power exceeds the lasing thresholds of 18.8, 86.1 and 30.3 mW, respectively. The slope efficiencies of 6.2%, 14.2% and 15.2% have been obtained from the linear fit of experiment data, respectively. As the pump power rises linearly to the maximum of 1006.2 mW, the output power of the pulsed waveguide laser reaches the maximum value of 66.7, 152.2 and 161.9 mW, respectively. Whilst for TM polarization (see Fig. 3(b)), the lasing thresholds measured in the experiment are 22.7, 90.8 and 53.0 mW in the VO_2_, graphene, and WS_2_ based systems, respectively. By the increase of launched power from the lasing threshold to the maximum value of 1006.2 mW, the Q-switching operates pulsed lasers with the maximum output power of 58.8, 136.8 and 141.1 mW and slope efficiencies of 5.9%, 12.8% and 14.6% for the three different SAMs, separately. According to Fig. 3, one could conclude that the pulsed waveguide laser performance in terms of lasing threshold, slope efficiency and maximum output laser at TE polarization is better than those at TM polarization for all of them. This may be partly due to the polarization properties on guidance of the superficial cladding waveguide in Nd:YVO_4_ crystal (Fig. 2). In addition, as one can see, with the phase-change material of VO_2_, the lasing threshold of the pulsed laser is much lower in comparison to those based on 2D materials. This is in good agreement with the Nd^3+^ doped vanadate bulk laser systems^44^. Under this condition, it becomes more easily to realize the pulsed laser generation with low intra-cavity intensity, indicating the sensitive nonlinear responses and revealing the promising application in optoelectronic sensors^46^,^50^. Moreover, in the aspect of maximum output power and slope efficiency, Q-switched waveguide laser with WS_2_ SAM has the highest value among three nanomaterials based systems, which is still feasible to reach higher-power output by increasing the pump power. For VO_2_, the phase-change material, it may be transferred from pulsed regime to CW operation as the temperature of the system exceeds the VO_2_ phase transition point due to the high-power laser induced heating effect^45^.

**Figure 4:**
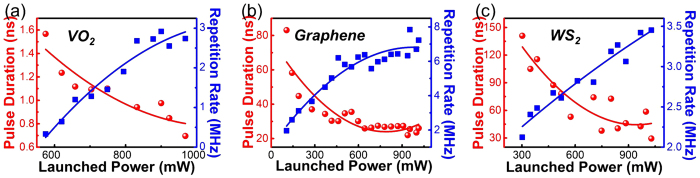
The pulsed laser properties with different SAMs. The pulse duration (red line) and repetition rate (blue line) of passively Q-switched waveguide laser as functions of launched pump power by using (**a**) VO_2_, (**b**) graphene and (**c**) WS_2_ as SAMs at TE polarization, respectively.

As to further investigate the pulse features of Q-switched waveguide laser based on the different SAMs, the minimum pulse durations as well as the maximum repetition rates and highest peak powers are measured at TE and TM polarization, respectively. For more direct comparison, the performance of Q-switched waveguide lasers in Nd:YVO_4_ crystal with three different SAMs are listed in Table 1. For brevity, the laser details are only depicted along TE polarization in Figs 4(a), (b) and (c), which demonstrate the dependences of pulse duration (red line) and repetition rate (blue line) as a function of launched power based on VO_2_, graphene and WS_2_ SAMs, respectively. With the increase of the pump power, the pulse duration values decrease correspondingly whilst the repetition rates present the uptrend on the whole, which is typical and reasonable for passively Q-switched lasers^51^. By varying the launched power, the repetition rates are tunable in a range from 0.3 to 2.9 MHz for VO_2_, 2.0 to 7.8 MHz for graphene and 2.1 to 3.5 MHz for WS_2_ SAMs, respectively. It is found that graphene based laser pulse has the highest repetition rate and broadest tunable range, which is proportional to single pulse energy and indicates the potential application of communications^52^,^53^. Simultaneously, the pulse durations turn from 1.57 to 0.69 ns with VO_2_, 83 to 22 ns with graphene and 141 to 39 ns with WS_2_ film, respectively, which presents that the laser pulse on account of VO_2_ SAMs has the shortest pulse duration of 690 ps in the subnanosecond scale with the highest peak power of 33.1 W. According to the following expression


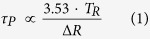


where *τ*_*P*_ is the pulse duration, *T*_*R*_ is the cavity round-trip time and *ΔR* is the maximum modulation depth^5^. It can be found that the pulse duration is inversely proportional to the maximum modulation depth. In addition, it is easier to get a higher maximum modulation depth than other SAs under the same pumping power due to the lower saturable intensity of VO_2_ film^37^. Based on the relative high modulation depth, shorter pulses obtained with VO_2_ are reasonable. Compared with the reported value of 14-nm pulse duration in the Q-switched erbium doped glass laser by utilizing the phase transition of a VO_2_ film^54^, the Q-switched Nd:YVO_4_ waveguide laser shows superior properties in the pulse duration based on VO_2_ SAM in the insulating phase. It is worth mentioning that the 690-ps waveguide laser is realized at room temperature with the insulting phase of VO_2_, which has simpler and easily manipulation than the reported 700-ps waveguide laser generation during the transition from insulating to metallic phase of VO_2_^45^. Moreover, based on so-called Contrast Nonlinear Transmission, much shorter pulse duration could be implemented during the ultrafast phase transition process of VO_2_.

**Figure 5:**
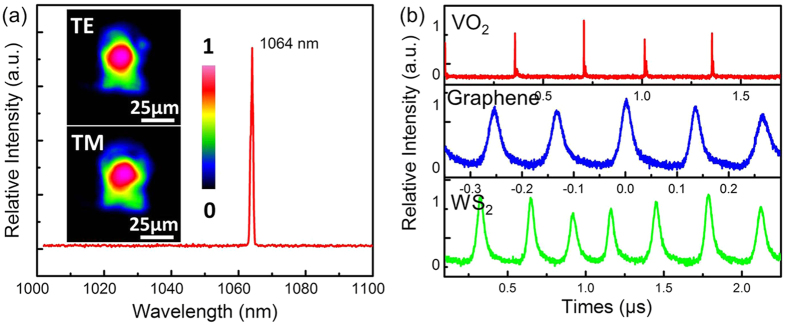
The emitted wavelength and pulse trace from the superficial cladding waveguide with three SAMs. Fig (**a**) Laser emission spectrum of the output pulsed laser from the Nd:YVO_4_ superficial cladding waveguide. The inserts display the laser modal profiles of TE_00_ and TM_00_ mode. (**b**) The typical oscilloscope traces of the Q-switched pulse with VO_2_ (red line), graphene (blue line) and WS_2_ (green line) as SAMs.

In VO_2_ based laser system, the spectrum of output laser, centering at a wavelength of 1064 nm above the threshold of pulsed waveguide laser, is depicted in Fig. 5(a), which corresponds to the main emission line of Nd^3+^ ion fluorescence transition band ^4^F_3/2_ → ^4^I_9/2_. In case of graphene and WS_2_ SAMs, the same laser spectra have been achieved. The insert images are the near-field modal distributions of the pulsed waveguide laser for the TE and TM polarization from the surficial cladding waveguide in Nd:YVO_4_ crystal. It can be easily observed that the main energy of the light fields is confined in the fundamental modes, which confirms the well-confined guidance of the emission pulsed laser. Figure 5(b) presents the pulse trains of the waveguide laser under the launched pump power at 968.3 mW by using SAMs of VO_2_, graphene and WS_2_ film, respectively. The pulse trains display the efficient Q-switched laser generation from superficial cladding waveguide.

In conclusion, the VO_2_ based Q-switched subnanosecond laser has been realized at room temperature in Nd:YVO_4_ waveguide fabricated by femtosecond laser writing. The pulsed lasers have also been implemented in the same waveguide platform by using graphene and WS_2_ thin film as SAMs. The lowest lasing threshold of ~19 mW and the shortest pulse duration of 690 ps have been achieved in the VO_2_ based system, whilst the maximum output power of ~162 mW and the highest repetition rate of ~7.8 MHz have been realized with WS_2_ and graphene SAMs, separately. These distinct laser performances have shown their respective features of three different SAMs for the fabrication of miniature pulsed laser sources.

## Methods

### Fabrication of superficial cladding waveguide

The laser-written waveguide with superficial cladding geometry is fabricated in an optically polished Nd:YVO_4_ crystal, which is cut with dimension of 10 mm × 5 mm × 2 mm, by using the laser facility of the Universidad de Salamanca, Spain. An amplified Ti:Sapphire femtosecond-laser (Spitfire, Spectra Physics) is employed as the laser source, delivering linearly-polarized pulses with a temporal duration of 120 fs at a central wavelength of 795 nm and operating at a repetition rate of 1 kHz. The maximum available pulse energy is 1 mJ, and it is tuned by a calibrated neutral density filter placed after a set of half-wave plate and a linear-polarization cube. The beam is then focused through one of the 10 mm × 5 mm sample surfaces by a 40 × microscope objective (N.A. = 0.4) and different pulse energies are tested, finding an optimum value at 0.21 μJ for fabricating the waveguide. During the writing process of the superficial cladding waveguide, the sample is mounted on a computer-controlled 3D-motorized stage, and scanned at constant velocity of 500 μm/s along the 10 mm axis, forming the damage track inside the sample. The procedure is repeated at different positions of the sample from bottom to top (so as to avoid the shielding of the incident pulses by the previously written damage tracks), following the desired major arc geometry with a lateral separation of 3 μm between each two adjacent damage tracks. Under this condition, the superficial cladding waveguide with the diameter of 50 μm is achieved. The inset of Fig. 1(a) depicts the optical microscope cross-sectional image of waveguide, which is 200 × magnified and imaged by an optical microscope (Axio Imager, Carl Zeiss) operating in the transmission mode.

### Characterization of Guidance and fluorescence

In order to characterize the waveguide, the fluorescence (related to Nd^3+^ ion emissions under the ^4^F_3/2_ → ^4^I_9/2_ and ^4^F_3/2_ → ^4^I_11/2_ transitions) and guidance properties have been investigated. On one hand, a confocal microscope system is utilized to investigate the micro-fluorescence properties with the purpose of studying the nature and spatial location of femtosecond-laser irradiated regions as well as exploring the potential application of the obtained waveguide as integrated laser gain medium. As the excitation source at the wavelength of 500 nm, a frequency-doubled Ti:Sapphire femtosecond laser (Maitai HP, Spectra-Physics) generates the laser pulses at a repetition rate of 80 MHz, which is focused by a 100 × microscope objective afterwards. The subsequent backscattered radiation signals generated by Nd^3+^ ions are collected by the same microscope objective and spectrally analyzed by a spectrometer (MS 3504i, SOLAR TII) equipped with a CCD detector (Andor, IDUS DU420A-OE) after passing through a set of filters, lenses and pinholes. The crystal is located on a 2D motorized stage to acquire the point-to-point emission spectra. On the other hand, the waveguiding experiment is performed by a typical end-face coupling arrangement with a 1064-nm solid-state laser which emits linearly polarized CW as the light source. After passing through the half-wave plate which can be rotated to change the polarization, the light is focused by one 20 × microscope objective to be coupled into one end-face of the waveguide, and then collected by another 20 × objective from output facet of the waveguide. The polarization guiding properties are characterized by a powermeter with converting the polarization of input light. In addition, the propagation losses of superficial cladding waveguide are determined by directly measuring the power of incident and output laser, taking the coupling efficiency and the Fresnel reflections in the interfaces of the waveguide end face and the air into account.

### Q-switched waveguide laser generation

For the room-temperature Q-switched waveguide laser generation, the VO_2_ film is coated on an MgF_2_ crystal by the pulsed deposition technique (PLD), acting as the saturable absorber mirror (SAM). The MgF_2_ crystal is with the dimension of 10 × 10 × 1 mm^3^, which is processed along the <110> direction, and the facets perpendicular to the <110> direction are polished. Furthermore, for the comparison with the VO_2_-based Q-switched laser performance, the commercial multilayer graphene (3~5 layers) and WS_2_ film, which are manufactured by chemical vapor deposition (CVD) on copper and nickel disks, and subsequently transferred to the polished surface of silica glass, are chosen as the SAMs. As shown in Fig. 1(a), the pulsed laser operation experiment is implemented by using an end pumping system with a linearly polarized beam at 808 nm, generated from a tunable CW Ti:Sapphire (Coherent MBR-PE) laser, as the laser source. A half-wave plate is employed to control the polarization of the pump laser, and the beam is converged to couple into the waveguide by a spherical convex lens with a focal length of 25 mm. The laser oscillation cavity used in this work adopts a typical Fabry-Perot configuration with the resonator mirrors closely attached the polished end-face of the waveguide. The input dielectric mirror has a high transmission of 98% at 808 nm and high reflectivity of 99% at 1064 nm, while the different saturable absorbers (VO_2_, multilayer graphene and WS_2_ film) are set as the output coupler mirrors (i.e., SAMs). The emitted pulse lasers are collected by a 20 × microscope objective (N.A. = 0.4) from the output surface of the waveguide and imaged by an infrared CCD camera. The emission laser spectra are analyzed by a spectrometer with resolution of 0.4 nm and a powermeter for 1064 nm is utilized for measuring the average output powers.

## Additional Information

**How to cite this article:** Nie, W. *et al*. Room-temperature subnanosecond waveguide lasers in Nd:YVO_4_ Q-switched by phase-change VO_2_: A comparison with 2D materials. *Sci. Rep.* **7**, 46162; doi: 10.1038/srep46162 (2017).

**Publisher's note:** Springer Nature remains neutral with regard to jurisdictional claims in published maps and institutional affiliations.

## Figures and Tables

**Table 1:** Comparison of Q-switched Nd:YVO_4_ waveguide lasers with different saturable absorbers.

Parameter	VO_2_		Graphene		WS_2_	
	TE	TM	TE	TM	TE	TM
**Lasing threshold (mW)**	18.8	22.7	86.1	90.8	30.3	53.0
**Output power (mW)**	66.7	58.8	152.2	136.8	161.9	141.1
**Pulse duration (ns)**	0.69	0.71	22	30	39	51
**Repetition rate (MHz)**	2.9	2.7	7.8	5.3	3.5	2.3
**Peak power (W)**	33.1	31.2	0.9	0.9	1.2	1.2
